# Prognostic value of [18F]-FDG PET/CT in patients with meta-static breast cancer treated with cyclin-dependent inhibitors

**DOI:** 10.3389/fonc.2023.1193174

**Published:** 2023-07-14

**Authors:** Alessio Annovazzi, Sandra Rea, Daria Maccora, Laura Pizzuti, Gianluigi Ferretti, Patrizia Vici, Federico Cappuzzo, Rosa Sciuto

**Affiliations:** ^1^ Nuclear Medicine Unit – IRCCS, Regina Elena National Cancer Institute, Rome, Italy; ^2^ Division of Medical Oncology 2 – IRCCS, Regina Elena National Cancer Institute, Rome, Italy; ^3^ Division of Medical Oncology 1 – IRCCS, Regina Elena National Cancer Institute, Rome, Italy; ^4^ Phase IV Clinical Trial Unit – IRCCS, Regina Elena National Cancer Institute, Rome, Italy

**Keywords:** PET-CT scan, fluorodeoxyglucose F18, breast cancer, cyclin-dependent kinase 4, progression-free and overall survival

## Abstract

**Objective:**

The addition of cyclin-dependent kinase 4/6 inhibitors (CDK4/6i) to endocrine therapy impressively improved the outcome of patients with hormone receptor-positive metastatic breast cancer. Despite their great efficacy, not all patients respond to treatment and many of them develop acquired resistance. The aim of this retrospective study was to assess the role of [18F]-FDG PET/CT in predicting PFS and OS in breast cancer patients treated with CDK4/6i.

**Methods:**

114 patients who performed an [18F]-FDG PET/CT scan before (PET1) and 2-6 months (PET2) after starting treatment were retrospectively enrolled. Metabolic response was evaluated by EORTC, PERCIST and Deauville Score and correlated to PFS and OS.

**Results:**

In patients who did not progress at PET2 (n = 90), PFS rates were not significantly different between classes of response by EORTC and PERCIST. Conversely, patients showing a Deauville score ≤3 had a longer PFS (median PFS 42 vs 21.0 months; p = 0.008). A higher total metabolic tumor volume at PET1 (TMTV1) was also associated with a shorter PFS (median 18 vs 42 months; p = 0.0026). TMTV1 and Deauville score were the only independent prognostic factors for PFS at multivariate analysis and their combination stratified the population in four definite classes of relapse risk. Conversely, the above parameters did not affect OS which was only influenced by a progressive metabolic disease at PET2 (3-years survival rate 29.8 vs 84.9%; p<0.0001).

**Conclusion:**

TMTV and metabolic response by Deauville score were significant prognostic factors for PFS in patients with breast cancer treated with CDK4/6i. Their determination could help physicians to select patients who may need a closer follow up.

## 1 Introduction

Breast cancer is the most common malignancy in women and a leading cause of cancer-related death (1). Approximately 70% of breast neoplasms express estrogen (ER) and/or progesterone (PR) receptors. Endocrine therapy (ET) is an effective treatment in hormone receptor (HR) positive HER2 negative advanced breast cancer (ABC), even if approximately 50% of patients develop resistance to anti-estrogen therapy within their lifetime (2). The development of endocrine resistance in breast cancer is usually associated with the deregulation of the cyclin-dependent kinase 4 (CDK4) and CDK6/retinoblastoma pathway. The recent introduction of CDK4/6 inhibitors (palbociclib, ribociclib, abemaciclib) to ET have impressively improved the outcome of patients with HR+ ABC, by overcoming some aspects of endocrine resistance (3–5). In *de novo* patients, as well as in second and later lines of treatment, all the CDK4/6 inhibitors performed better than ET alone in terms of selected clinical outcome. In a recent meta-analysis (6), Munzone et al. reported that the combination of CDK4/6i and ET is superior to ET alone in terms of PFS and OS, regardless of the drug administered, treatment line, age distribution, race, progesterone receptor status, menopausal status, site of metastasis and endocrine resistance status. Moreover, the three CDK4/6 inhibitors (CDK4/6i) reported a good toxicity profile, without any difference in terms of progression-free survival (PFS) for patients who needed a dose reduction due to adverse events compared with those who did not. However, intrinsic or acquired resistance can cause disease progression in a large number of patients and the understanding of the mechanism of resistance is an urgent clinical need (7, 8). In breast cancer, PET/CT with [18F]-FDG is routinely used in the initial staging of patients with locally advanced neoplasms candidates for neoadjuvant chemotherapy, as well as for the response evaluation to chemotherapy and hormonal treatment in patients with ABC. Several studies have shown the effectiveness of [18F]-FDG PET/CT in assessing response to systemic therapy in ABC (9–12), nevertheless there is little scientific evidence on its specific use in evaluating treatment response of cyclin inhibitors, often limited to case reports. In a pilot study on 12 patients treated with palbociclib, Taralli et al. (13) demonstrated that PET with FDG allowed to correctly reclassify 8 patients judged with conventional imaging as stable disease, with disease progression in 3 and partial or complete metabolic response in 5. It is not known whether the depth of metabolic response to treatment (stable metabolic disease, partial or complete metabolic response) correlates with PFS. Two standard semi-quantitative PET response criteria in solid tumors, the European Organization for Research and Treatment of Cancer (EORTC) criteria (14) and PET Response Criteria in Solid Tumors (PERCIST) (15) are available. Deauville visual response criteria (16, 17) were specifically developed for lymphoma where it is currently used in daily routine and clinical trial, but there is increasing evidences concerning its use in solid tumors, like in lung cancer (18) and head and neck tumors (19). In this study we compared the EORTC, PERCIST, and Deauville criteria as well as absolute value and variation of standard PET metrics for response assessment during therapy with CDK4/6i to evaluate the most effective to predict PFS and overall survival (OS). We also investigated the possible role of pre-treatment Total Metabolic Tumor Volume (TMTV), a surrogate for tumor burden, and whole-body Total lesion glycolysis (TTLG), together with clinic-pathologic tumor variables in predicting response to treatment and PFS or OS.

## 2 Materials and methods

### 2.1 Patients

The study has been approved by the local institutional ethics committee (Prot. no. 1678/22) and has been performed in compliance with the ethical standards. Given the retrospective design of the study, the ethics committee allowed the use and processing of the patient clinical data even in the absence of written informed consent for all patients who gave permission to use their data in anonymous and aggregate form for research activities. A database search was performed for patients with metastatic breast cancer who started treatment with CDK4/6i between January 2017 and August 2021 who performed an [18F]-FDG PET/CT scan at our Institute. Inclusion criteria of this study were: (a) availability of an [18F]-FDG PET/CT before treatment (PET1) showing at least one disease localization with significant FDG uptake; (b) a second follow-up [18F]-FDG PET/CT scan (PET2) had to be performed after at least 2 months and no later than 6 months after the beginning of treatment. A total of 133 patients were initially identified, 19 of them were excluded from the analysis for insufficient follow-up data or for not fulfilling inclusion criteria. A final cohort of 114 patients was finally analyzed. The median age of the 114 patients was 59 years (interquartile range, IQR 51-72 years). The most frequent disease sites at PET1 were bone (81.1%) and lymph nodes (53.6%). All of the patients received a combination of aromatase inhibitor or fulvestrant and a CDK4/6 inhibitor (palbociclib n = 57; ribociclib n = 45; abemaciclib n = 12), 70 of them (61.4%) as first-line treatment. Considering patients treated in the second line, 74% had received hormonal therapy while 26% chemotherapy in the first-line setting. More specifically, as regards the patients who had received first-line hormone therapy, 81% had received aromatase inhibitor monotherapy. The remaining patients received fulvestrant or everolimus in combination with exemestane. The baseline characteristics of the 114 patients enclosed in the analysis are detailed in **Table 1**. Patient clinical outcome was measured by PFS and OS, defined as the period starting from the date of first treatment and disease relapse or death, respectively. During an estimated median follow-up of 35.8 months, 69 out of 114 patients (60.5%) showed disease progression. Overall, 66.2% and 41.9% of patients were progression free at 12 and 36 months, respectively, with a median PFS of 24.6 months (95% CI 16.6-37.2 months). Median time from start of treatment to response assessment with PET/CT was 4.6 months (IQR 3.7–5.3 months). The median survival time was 63.9 months with a 3-years survival rate of 72.9%.

**Table 1:** General Characteristics of Patients, Tumor and treatment.

Characteristics	Data
**Age (years), median (IQR)** **Age > 60 years**	59 (51–72) n= 54 (47.4%)
**ECOG Performance status** **•0** **•1** **•NA**	n = 78 n = 24 n = 12
**Progesterone receptor status** **•Positive** **•Negative** **•NA**	n = 88 n = 11 n = 15
**Primary tumor** **•Luminal A** **•Luminal B** **•NA**	n= 26 n= 70 n= 18
**Disease location** **•Bone** **•Lymph node** **•Liver** **•Lung/pleura** **•Locoregional** **•others**	n= 71 (81.1%) n= 61 (53.6%) n= 19 (16.7%) n= 13 (13.4%) n= 30 (26.3%) n= 2 (1.8%)
**Number of metastatic sites** **•1** **•>1**	n= 62 (54.4%) n= 52 (45.6%)
**CDK4/6 inhibitor** **•Palbociclib** **•Ribociclib** **•Abemaciclib**	57 (50%) 45 (39.5%) 12 (10.5%)
**CDK4/6 inhibitor therapy line** **•1^st^ line** **•> 1^st^ line**	n=70 (61.4%) n=44 (38.6%)

IQR, Interquartile range**;** NA, not available; ECOG, Eastern Cooperative Oncology Group.

### 2.2 [18F]-FDG PET/CT acquisition and image analysis

A combined PET/CT imaging was performed using a Siemens Biograph 16 (Siemens Healthineers). Patients fasted for a minimum of 6 hours before the scan and glucose levels below 150 mg/dl were required at the time of tracer injection. PET/CT acquisition was performed 60 ± 10 min. after intravenous injection of an average dose of 5 MBq/kg of [18F]-FDG. A non-contrast-enhanced whole-body CT scan was acquired for anatomic localization and attenuation correction of PET images. The following parameters were used: 120-140 Kev, 4 mm slice thickness using “CAREDose” software to reduce radiation dose and optimize image quality. PET data were acquired on a 3D mode immediately after the CT scan 2-3 minutes for each bed position. PET images were reconstructed by an ordered subset expectation maximization (OSEM) algorithm (TrueX, Siemens Healthineers) with point spread function modelling (3 iterations, 21 subsets). After reconstruction, the images were filtered by a Gaussian filter with a full width at half maximum of 4 mm. [18F]-FDG PET/CT data images were reviewed using a dedicated workstation (Syngo.via, Siemens Healthineers). TMTV was computed using the vendor-supplied automatic analysis package, as the sum of the metabolic volumes of all pathologic lesions having a SUVmax >2.5 and a volume >0.5 cm3. TLG was calculated as the product of SUVmean and MTV and TTLG as the sum of TLG of all pathologic lesions. Foci not automatically included were added manually and areas of physiologic FDG uptake were excluded by an experienced nuclear medicine physician. TMTV, TTLG and SUVmax of the hottest lesion at PET1 (TMTV1, TTLG1 and SUV1, respectively), absolute value of TMTV (TMTV2), TTLG (TTLG2) and SUVmax at PET2 (SUV2) as well as their percentage change between PET2 and PET1 (ΔSUV, ΔTMTV, ΔTTLG) were also calculated.

### 2.3 Assessment of tumor response

We selected up to 5 of the lesions with the highest FDG uptake (up to two lesions per organ) at PET1 and measured the same lesions on the subsequent follow-up scan. Metabolic response was evaluated according to EORTC (14), PERCIST1 (analyzing SUVpeak of the hottest lesion) (15) and PERCIST5 (analyzing the SUVpeak of 1 or up to 5 lesions) (20), classifying the patients into 4 response groups: complete metabolic response (CMR), partial metabolic response (PMR), stable metabolic disease (SMD), and progressive metabolic disease (PMD). The Deauville 5-points score was evaluated considering the hottest lesion at PET2 as follow: score 1, no FDG uptake (same as background); score 2, uptake ≤ than that of mediastinal blood pool; score 3, uptake higher than mediastinal blood pool but ≤ than that of liver; score 4, uptake moderately higher than that of liver; score 5, uptake markedly stronger than that of liver. Study patients were then divided in two classes as for responder (score 1-3) and non-responders (score 4 or 5) in lymphoma. A sub-analysis in three classes (score 1-2, 3 and 4-5) was also performed. For each patient, response criteria were reported by one of three experienced nuclear medicine physicians (AA, DM and SR) who reviewed [18F]-FDG PET/CT images and who were unaware of the patient clinical outcome at the time of image analysis.

### 2.4 Statistical analysis

Statistical analyses were performed by R software (ver. 4.1.0). A p value <0.05 was considered statistically significant for type 1 error. Differences among PET and clinical parameters vs response to therapy were assessed by Mann-Whitney U test for quantitative variables (Age, TMTV1, TTLG1) and by Fisher’s exact test for qualitative or quantitative variables after dichotomization (TMTV1, TTLG1). Concordance between EORTC and PERCIST were assessed using Cohen’s k-coefficients. Progression-free Survival (PFS) and overall survival (OS) were calculated from the date of starting CD4/6i to the date of disease progression or death, respectively. For PFS estimation, only parameters of patients not showing a PMD at PET2 were included in the analysis. All PET quantitative calculated parameters were dichotomized for survival analysis using the Maximally selected rank statistics (Package ‘maxstat’ in R). PFS and OS curves of each response criteria and PET metrics were estimated by the Kaplan–Meier methods and tested for significance using the log-rank test. Clinical and tumor parameters possibly affecting PFS or OS were also analyzed. A Cox proportional-hazards univariate regression analysis was also performed to determine hazard ratios (HR) of predictive factors for PFS and OS. A multivariate Cox proportional hazard model was then created using stepwise selection of statistically significant (p< 0.05) variables in the univariate model.

## 3 Results

### 3.1 PET/CT response assessment

A high agreement was observed between EORTC criteria and PERCIST5 (k = 0.95), slightly lower for EORTC vs PERCIST1 (k = 0.83) and PERCIST1 vs PERCIST5 (k = 0.86). According to EORTC, PERCIST1 and PERCIST5, PMR were reported in 59, 52 and 57 patients, SMD in 10, 17 and 12 patients, respectively (**Table 2**). Patients with PMD (n = 24) and CMR (n = 21) were correctly identified with all the semi-quantitative criteria and by Deauville visual score. Among patients with no PMD at PET2 (n = 90), the number of patients with Deauville scores of 1-5 were 21 (23.3%), 15 (16.7%), 25 (27.8%), 23 (25.6%), and 6 (6.7%), respectively (**Table 2**). All patients with PMD had Deauville score 5. None of clinical and PET parameters were predictive for treatment response at PET2.

**Table 2:** Cox proportional hazard regression models for PFS and OS fitted by the response criteria (EORTC and PERCIST) and by Deauville score in patients with no PMD at PET2.

criteria	response class	N	PFS	p	OS	p
			HR (95% CI)		HR (95% CI)	
**EORTC**	CMR PMR SMD	**21** **59** **10**	1 1.68 (0.77-3.6) 1.70 (0.55-5.2)	0.20 0.37	1 0.79 (0.21-3.07) 1.46 (0.24-8.85)	0.74 0.68
	CMR Non-CMR	**21** **69**	1 1.68 (0.78-3.62)	0.19	1 0.88 (0.24-3.26)	0.85
**PERCIST1**	CMR PMR SMD	**21** **52** **17**	1 1.86 (0.85-4.07) 1.12 (0.39-3.24)	0.12 0.84	1 1.04 (0.28-3.91) 0.40 (0.04-3.87)	0.96 0.43
	CMR Non-CMR	**21** **69**	1 1.68 (0.78-3.62)	0.19	1 0.88 (0.24-3.26)	0.85
**PERCIST5**	CMR PMR SMD	**21** **57** **12**	1 1.65 (0.75-3.62) 1.82 (0.66-5.0)	0.21 0.25	1 0.85 (0.22-3.27) 1.05 (0.17-6.34)	0.81 0.96
	CMR Non-CMR	**21** **69**	1 1.68 (0.78-3.62)	0.19	1 0.88 (0.24-3.26)	0.85
**Deauville score**	1-2 3 4-5	**36** **25** **29**	1 1.77 (0.8-3.9) 2.88 (1.38-6.03)	0.16 **0.005**	1 1.59 (0.35-7.14) 2.01 (0.48-8.46)	0.55 0.34
**Deauville score**	≤3 >3	**61** **29**	1 2.19 (1.21-3.96)	**0.009**	1 1.61 (0.36-7.26)	0.54

HR, hazard ratios; CI, confidence intervals.

Bold values are statistically significant (<0.05).

### 3.2 Progression-free survival

**Figure 1:**
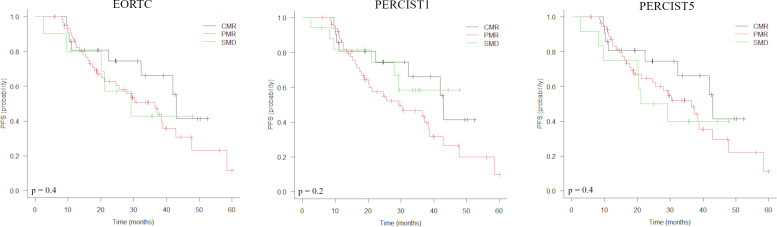
Kaplan-Meier estimations of progression-free survival (PFS) accordingly to EORTC, PERCIST1 and PERCIST5 response criteria.

Baseline characteristics associated with a shorter PFS were administration of CDK4/6i therapy in second- or later line, liver metastases, bone metastases and TMTV1 >31.4 cm3 (**Table 3**). In the sub-group of responders at PET2 (n= 90), Deauville score >3 was associated with a shorter PFS. Metabolic response classes according to EORTC/PERCIST (**Table 2**, **Figure 1**), values of TMTV2 as well as ΔTMTV and ΔSUVmax were not predictive for PFS. In the multivariate analysis, only TMTV1 >31.4 cm3 and Deauville score >3 were the independent prognostic factors for PFS (**Table 3**). PFS was significantly longer in patients with TMTV1 <31.4 cm3 (3-years progression-free rate of 61 vs 22.7%; median PFS 42 vs 18 months; p = 0.0026) as well as in patients with a Deauville score ≤3 at PET2 (3-years progression-free rate 65.2 vs 29.3%; median PFS 42 vs 21 months; p = 0.008). There was a trend toward a shorter PFS for patients with Deauville score 3 vs those with score 1-2, although not statistically significant (p = 0.1). TTLG predicted PFS slightly worse than TMTV, reaching only the limit of statistical significance (p = 0.12).

**Table 3:** Univariate and multivariate Cox proportional-hazards regression for clinical and PET parameters and the prediction of PFS and OS.

Univariate analysis	PFS		OS	
Clinical Parameter	HR (95% CI)	p	HR (95% CI)	p
**Age** **Age >60 years**	0.99 (0.98-1.01) 0.85 (0.52-1.38)	0.53 0.49	1.01 (0.98-1.04) 1.15 (0.57-2.34)	0.58 0.7
**ECOG**	1.01 (0.56-1.80)	0.98	2.28 (1.04-5.0)	**0.04**
**Luminal-B**	0.96 (0.56-1.66)	0.89	1.46 (0.61-3.52)	0.4
**Pg receptor positive**	1.01 (0.46-2.25)	0.97	0.88 (0.26-2.93)	0.83
**CDK4/6 inhibitor >1^st^ line**	1.92 (1.2-3.0)	**0.008**	2.27 (1.1-4.7)	**0.03**
**Liver metastases**	2.0 (1.1-3.6)	**0.02**	3.1 (1.4-6.8)	**0.005**
**Bone metastases**	1.95 (1.08-3.52)	**0.03**	1.93 (0.74-5.02)	0.18
**Lung/pleural metastases**	1.39 (0.66-2.94)	0.39	0.7 (0.17-2.94)	0.63
**N° of metastatic sites >1**	1.09 (0.67-1.76)	0.74	**1.09 (0.54-2.22)**	0.81
PET Parameter				
**TMTV1 >31.4 cm^3^**	2.11 (1.28-3.45)	**0.003**	1.3 (0.64-2.65)	0.46
**TTLG1 > 145**	1.74 (0.87-3.47)	0.12	1.36 (0.6-3.06)	0.46
Deauville score >3^*^	2.19 (1.21-3.96)	**0.009**	1.58 (0.5-5)	0.43
**Deauville score (all)**	–	–	4.78 (2.06-11.1)	**<0.001**
**PMD at PET2**	–	–	10.7 (5.0-22.6)	**<0.001**
Multivariate analysis				
**TMTV1 >31.4 cm^3^**	3.83 (1.94-7.57)	**<0.001**	–	–
Deauville score >3^*^	1.95 (1.06-3.58)	**0.03**	–	–
**PMD at PET2**	–	–	9.82 (4.62-20.9)	**<0.001**
**Liver metastases**	–	–	2.34 (1.06-5.18)	**0.04**

^*^Only patients with no PMD at PET2 (n= 90).

Bold values are statistically significant (<0.05).

**Figure 2:**
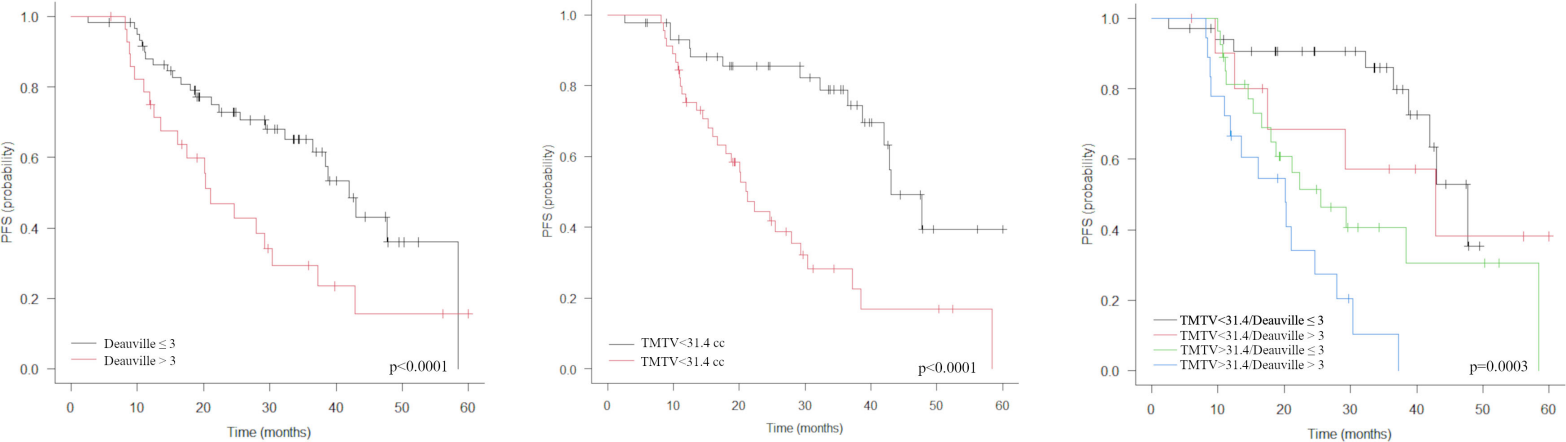
Kaplan-Meier estimations of progression-free survival (PFS) accordingly to Deauville score, TMTV (middle) and TMTV plus Deauville score (right). The combination of pre-treatment TMTV with treatment response by Deauville score allows to stratify the population into 4 distinct classes by risk of relapse (right).

**Figure 3:**
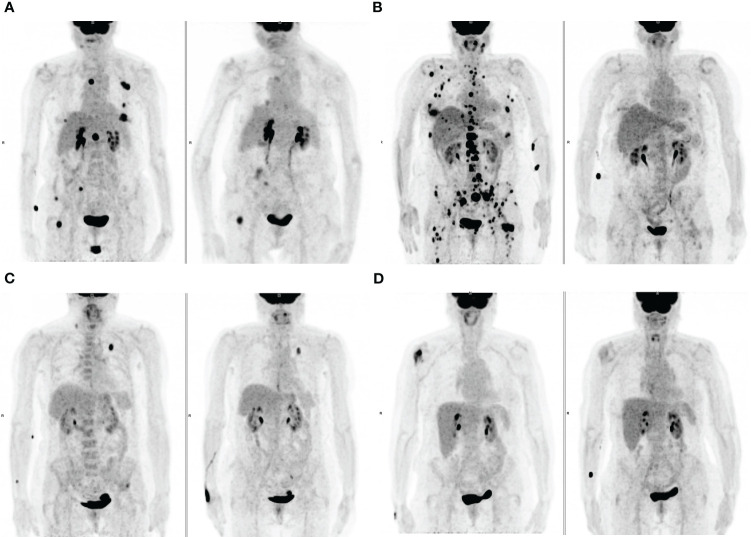
Maximum-intensity projection (MIP) FDG PET images at baseline (left) and at first PET evaluation during treatment (right). **(A)** An 83-years old patient with lymph node and bone metastases and a high TMTV1 (50.9 cm3) showing PMR at PET2 with a Deauville score >3 (5). **(B)** a 72-years old patient with lymph node, bone and liver metastases and a high TMTV1 (212.4 cm3) showing PMR at PET2 with a Deauville score ≤ 3 (3). **(C)** A 75-years old patient with metastases in the left lung, left iliac bone and in a right supraclavicular lymph node with low TMTV1 (8.3 cm3) showing PMR at PET2 with a Deauville score >3 (4). **(D)** A 73-years old patient with a single metastasis in the right humeral head with low TMTV1 (7.7 cm3) showing PMR at PET2 with a Deauville score ≤ 3 (3).

The combination of pre-treatment TMTV with treatment response by Deauville score stratifies the population into 4 distinct classes by risk of relapse (**Figures 2**, **3**): low TMTV1/Deauville ≤3 (3-years progression-free rate 85.9%; median PFS 47.7 months), low TMTV1/Deauville >3 (3-years progression-free rate 57.1%; median PFS 42.8 months), high TMTV1/Deauville ≤3 (3-years progression-free rate 40.6%; median PFS 25.5 months) and high TMTV1/Deauville >3 (3-years progression-free rate 10.2%; median PFS 20.1 months).

### 3.3 Overall survival

Baseline features associated with a shorter OS were ECOG status, administration of CDK4/6i therapy in second- or later line and liver metastases (**Table 3**); patients showing PMD at PET2 were associated with a significantly shorter OS (3-years survival rate 29.8 vs 84.9%, median OS 24 months vs not reached; p<0.0001).

**Figure 4:**
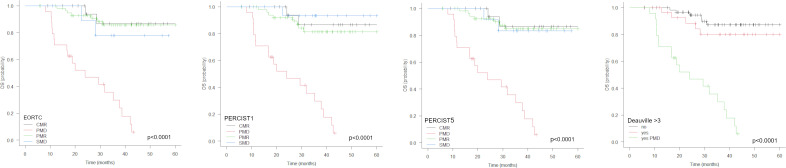
Kaplan-Meier estimations of overall survival (OS) accordingly to response criteria (EORTC, PERCIST1, PERCIST5) and Deauville score.

No significant differences were observed between the CMR, PMR and SMD groups with PERCIST1, PERCIST5 and EORTC, nor with Deauville score among patients with no PMD at PET2 (**Table 2**, **Figure 4**). By multivariate analysis, PMD at PET2 and liver metastases remained independent predictors of death.

## 4 Discussion

In the present study, we evaluated the role of [18F]-FDG PET/CT and compared the performance of response assessment by standard semi-quantitative criteria (EORTC and PERCIST) and by a visual scale (namely Deauville score) routinely used in lymphoma (16, 17) to predict PFS and OS in patients with metastatic breast cancer receiving therapy with CDK4/6i. It is well known that criteria based on tumor size modification are limited using target therapies, having a more cytostatic than a cytotoxic effect on cancer cells. Moreover, change in tumor size is not a good surrogate of bone lesion response (21), as a paradoxical increase in size and number at CT scan due to osteoblastic reaction in healing bone can be observed in responding lesions. The higher accuracy of [18F]-FDG PET/CT over conventional imaging (CT scan and bone scintigraphy) in monitoring response to therapy in metastatic breast cancer has been demonstrated in many studies (9). In an observational study, Hidlebrandt et al. (10) reported an improved patient management and a survival benefit of 14-24 months when [18F]-FDG PET/CT was used alone or in combination with CT, leading to a treatment change on average 5 months earlier. Nevertheless, some patients showed early disease progression and the prompt identification of non-responders’ patients represents an urgent clinical need.

Despite discrepancies in classifying PMR and SMD, we observed a good agreement among EORTC criteria and PERCIST, like reported in previous studied on different neoplastic conditions and summarized in a pooled analysis by Kim et al. (22). Nevertheless, metabolic response classes according to EORTC and PERCIST were not predictive for PFS, nor for OS. Surprisingly, patients showing CMR by EORTC or PERCIST did not show a prolonged PFS as compared to PMR and SMD. This atypical behavior observed herein differs from what has been reported in other studies. In a cohort of 57 patients with newly diagnosed metastatic breast cancer treated with first line systemic therapy, Depardon et al. (23) reported that CMR either with EORTC or PERCIST was an independent predictor of survival. Conversely, results of the present study showed that patients having a Deauville score ≤3 at PET2 showed a significantly longer PFS, and it was confirmed as an independent prognostic factor at multivariate analysis. It can be postulated that in this specific treatment setting, a significant reduction of FDG lesion uptake, like that observed for Deauville score ≤3 could be indicative of treatment response. After all, residual tumor uptake has been reported in patients with breast cancer achieving a pathologic complete response after neo-adjuvant chemotherapy (22). On the contrary, the persistence of a high FDG lesion uptake can reflect the onset of drug resistance. The above results suggest that the Deauville criteria, which represent the gold standard for response assessment in lymphomas (16, 17), can also be conveniently applied in this specific setting. In fact, there is growing interest in using qualitative scales to evaluate treatment response in solid tumors as well, particularly in assessing response to radiotherapy. Peter Mac (24) and Deauville score were significantly associated with OS and showed better efficacy than EORTC and PERCIST in distinguishing CMR and non-CMR in a cohort of 87 NSCLC patients undergoing radical chemoradiotherapy (18). In 114 patients affected by esophageal squamous cell carcinoma a Deauville adapted 4-point scale provided good predictive value for survival outcome, also yielding an excellent inter-observer agreement between reviewers from different centers (25). Another visual scales derived from the Deauville score, the Cuneo criteria, was suitable applied to evaluate response of radiotherapy in head and neck cancer (26). Moreover, the Deauville criteria have the undoubted advantage of reproducibility and widespread familiarity among nuclear medicine physicians.

In the present study, we also aimed to identify possible PET/CT metrics at pre-treatment scan affecting patient clinical outcome. Some characteristics like age, absence of progesterone expression, presence of liver metastases, line of treatment with CDK4/6i and best response achieved have been identified as the most predictive factors of the response to this treatment regimen and of the progression risk (27). In our analysis, CDK4/6i therapy as second line or beyond and liver metastases were associated with PFS, but only at univariate analysis. Among all pre-treatment PET and clinical parameters analyzed, TMTV, a surrogate marker for tumor burden, was the only parameter confirmed as independent prognostic factor at multivariate analysis, in addition to the category of metabolic response by Deauville score at PET2. According to some previous papers, TMTV has been already demonstrated as a strong prognostic imaging marker in other oncologic conditions (28–30). Moreover, from the combination of TMTV and Deauville score it was possible to stratify the study population into 4 relapse risk classes (**Figure 2**). Among them, TMTV has the greatest impact, as evidenced by the analysis of survival curves and by HR at multivariate analysis. Conversely, liver metastases, already reported as a poor prognostic factor in patients treated with CDK4/6i (31, 32), and PMD at PET2 were the only features affecting OS, while TMTV did not. It could be postulated that pre-treatment tumor burden may influence the likelihood of disease progression, while other factors more closely related to tumor biology, such as the ability to metastasize to the liver or resistance to cyclin treatment itself, may have a greater impact on survival.

Our study has some limitations. First, the retrospective design makes led to selection bias. Moreover, the accuracy of assessment of treatment response can be influenced by the high range time interval (2-6 months) between start of treatment and first follow-up PET/CT imaging. As only a few patients have performed a PET2 less than three months after the start of treatment, it is not possible to evaluate whether a PET scan performed earlier can provide overlapping results. Finally, different combinations of CDK4/6i and hormonal treatment were used in this cohort of patients. On the other hand, some strengths of the present study needed to be highlighted. The inclusion of 114 HR+ HER2-negative ABC participants makes our study the largest conducted thus far on this specific topic. Patients included in this analysis were treated in routine clinical practice, thus our results can be considered as perfectly consistent with the current therapeutic landscape. Another strength of the study is the remarkable duration of the follow-up (median of 26.6 months). In conclusion, [18F]-FDG PET/CT may have a key prognostic role in ABC patients treated with CDK4/6i. Determination of pre-treatment TMTV and metabolic response evaluation by Deauville score could help physicians to select those patients who may need a closer follow up. Further studies with a prospective design and more homogenous study population will be needed to confirm the prognostic value of the evaluated parameters in this subset of patients. 

## Data availability statement

The raw data supporting the conclusions of this article will be made available by the authors, without undue reservation.

## Ethics statement

The studies involving human participants were reviewed and approved by IRCCS – Regina Elena National Cancer Institute ethics committee. The ethics committee waived the requirement of written informed consent for participation.

## Author contributions

Conceptualization: AA and RS. Methodology: SR and DM. Supervision: RS and FC. Validation: AA, DM and SR. Formal analysis: AA and RS. Investigation: AA and RS. Resources: RS, LP, GF and PV. Data curation: DM and RS. Writing—original draft preparation: AA, DM and LP. Writing—review and editing: AA, SR, GF, PV, FC and RS. Visualization: SR and AA. All authors have read and agreed to the published version of the manuscript.
